# Activated MEK cooperates with *Cdkn2a* and *Pten* loss to promote the development and maintenance of melanoma

**DOI:** 10.1038/onc.2016.526

**Published:** 2017-03-06

**Authors:** H Yang, D A Kircher, K H Kim, A H Grossmann, M W VanBrocklin, S L Holmen, J P Robinson

**Affiliations:** 1 Hormel Institute, University of Minnesota, Austin, MN, USA; 2 Huntsman Cancer Institute, University of Utah Health Sciences Center, Salt Lake City, UT, USA; 3 Department of Oncological Sciences, University of Utah Health Sciences Center, Salt Lake City, UT, USA; 4 Department of Pathology, University of Utah Health Sciences Center, Salt Lake City, UT, USA; 5 ARUP Laboratories, Salt Lake City, UT, USA; 6 Department of Surgery, University of Utah Health Sciences Center, Salt Lake City, UT, USA

## Abstract

The development of targeted inhibitors, vemurafenib and dabrafenib, has led to improved clinical outcome for melanoma patients with BRAF^V600E^ mutations. Although the initial response to these inhibitors can be dramatic, sometimes causing complete tumor regression, the majority of melanomas eventually become resistant. Mitogen-activated protein kinase kinase (MEK) mutations are found in primary melanomas and frequently reported in BRAF melanomas that develop resistance to targeted therapy; however, melanoma is a molecularly heterogeneous cancer, and which mutations are drivers and which are passengers remains to be determined. In this study, we demonstrate that in BRAF^V600E^ melanoma cell lines, activating MEK mutations drive resistance and contribute to suboptimal growth of melanoma cells following the withdrawal of BRAF inhibition. In this manner, the cells are drug-addicted, suggesting that melanoma cells evolve a ‘just right’ level of mitogen-activated protein kinase signaling and the additive effects of MEK and BRAF mutations are counterproductive. We also used a novel mouse model of melanoma to demonstrate that several of these MEK mutants promote the development, growth and maintenance of melanoma *in vivo* in the context of *Cdkn2a* and *Pten* loss. By utilizing a genetic approach to control mutant MEK expression *in vivo,* we were able to induce tumor regression and significantly increase survival; however, after a long latency, all tumors subsequently became resistant. These data suggest that resistance to BRAF or MEK inhibitors is probably inevitable, and novel therapeutic approaches are needed to target dormant tumors.

## Introduction

The mitogen-activated protein kinase (MAPK) signaling pathway is constitutively activated in over 85% of malignant cutaneous melanomas, due to BRAF (~40%), NRAS (~25%), NF1 (~13%) and mitogen-activated protein kinase kinase (MEK) (~8%) mutations.^1, 2, 3^ BRAF^V600E/D/K^ mutations (*BRAF*^*T1799A/G/GTIndelAA*^) lead to constitutive kinase activity and elevated downstream signaling, which drives cell proliferation and survival; however, these mutations are also found in benign nevi, and alone are insufficient for malignancy.^4^ Homozygous deletion of cyclin-dependent kinase inhibitor 2A (*CDKN2A*), the melanoma susceptibility locus, is found in ~40% of sporadic melanomas; and promoter methylation leading to silencing and loss of expression is observed in a high proportion of the remaining tumors.^5^ This locus encodes two proteins, p16 (INK4a) and p14 (ARF). Loss of p14^ARF^ permits unregulated cell division while loss of p16^INK4a^ leads to dysregulation of p53 and pervasive genetic instability.^6, 7^

**Figure 1:**
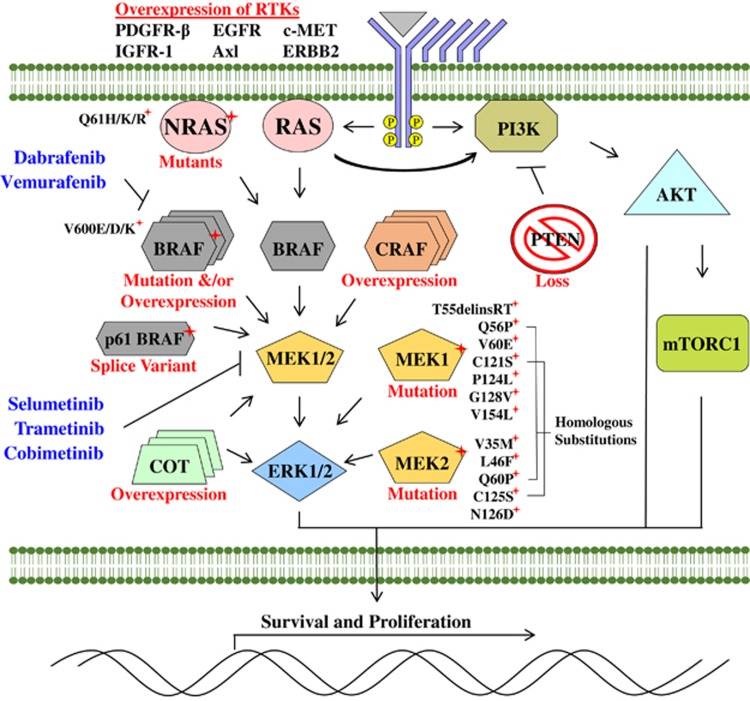
Scheme depicting mechanisms of resistance observed to BRAF and MEK1/2 inhibitors *in vitro* and *in vivo*. Resistance is largely mediated by alternative means of MAPK pathway activation. Mutations in MEK1 and MEK2 that interfere with drug binding-pockets or that upregulate inherent kinase activity mediate resistance to both BRAF and MEK inhibitors. The pathway can also be reactivated through gain-of-function NRAS^Q61H/K/R^ mutations, alternative splice variants of BRAF^V600E^, overexpression of BRAF^V600E^, CRAF or Cancer Osaka thyroid oncogene (COT1) or phosphatase and tensin homolog (PTEN) mutations. Overexpression of RTKs including platelet-derived growth factor receptor β (PDGFRβ), epidermal growth factor receptor (ERBB2), insulin-like growth factor 1 receptor (IGFR1), hepatocyte growth factor receptor (MET) and AXL RTK have also been proposed to drive resistance.

In patients with late-stage BRAF^V600E^ melanomas, BRAF inhibitors (for example, dabrafenib or vemurafenib) confer a survival advantage when compared with chemotherapy, demonstrating improvements in response-rates, progression-free survival and overall survival.^8, 9^ Initial responses to BRAF inhibitors are not durable, and patient relapse usually occurs within 6–7 months.^8, 10^ The use of concurrent BRAF and MEK inhibitors (for example, cobimetinib, selumetinib or trametinib) for patients with melanoma has been established as a synergistic treatment approach and one that has further improved response compared with BRAF monotherapy.^11^ However, the majority of patients still develop resistance^12, 13^ (Figure 1). Mechanisms of resistance to single agent or combination therapies include mutations in MEK1 (*MAP2K1*) and MEK2 (*MAP2K2*), increased COT1 (*MAP3K8*) and CRAF expression, NRAS-activating mutations, gain in BRAF^V600E^ copy number and splice variants impervious to mutant-specific BRAF inhibitors,^14, 15, 16, 17, 18, 19, 20^ and the up-regulation of the receptor tyrosine kinases (RTKs), including MET, AXL, ERBB2, PDGFR-β, EGRF and IGF1R. These RTKs can also activate the phosphatidyl-inositol-3-kinase/AKT pathway, which is active in most melanomas,^21^ due to the loss of PTEN expression, or activation of PI3KCA or AKT mutation.^22, 23, 24, 25, 26, 27^ A phase II clinical study by Trunzer *et al.*^28^ identified activating MEK1^Q56P^ and MEK1^E203K^ mutations in vemurafenib-resistant melanomas that were not present in pre-treated tumors. These mutations were predictably accompanied by a strong up-regulation of phosphorylated MAPK/extracellular signal-regulated kinase (ERK) levels, indicative of MAPK pathway stimulation.^28^ Similarly, Emery *et al.*^29^ detected a MEK1^P124L^ mutation that emerged in a resistant metastatic melanoma following patient treatment with PLX4720 (a BRAF inhibitor closely related to vemurafenib and selumetinib).^29^ A massively parallel sequencing study by Wagle *et al.*^30^ identified a MEK1^C121S^ mutation in a metastatic melanoma patient who had developed clinical resistance to vemurafenib. This mutation was later shown to confer robust resistance to PLX4720 and selumetinib *in vitro*.^11^ More recently, Van Allen *et al.*^14^ conducted a comprehensive whole-exome sequencing study of BRAF^V600E^-resistant metastatic melanomas from 45 patients who received vemurafenib or dabrafenib monotherapy. Numerous MEK mutations were detected in these tumors, including MEK1^V60E^, MEK1^G128V^, MEK1^V154L^, MEK2^V35M^, MEK2^L46F^, MEK2^C125S^ (homologous to MEK1^C121S^) and MEK2^N126D^, none of which had been detected in pre-treated tumors. *In vitro* experiments confirmed tumor resistance to dabrafenib and trametinib, but not to ERK inhibition.^14^ MEK1^T55IndelRT^ has also been reported to drive resistance in tumor xenografts.^31^ A meta-analysis by Johnson *et al.* described an overall incidence of 7% for MEK1/2 mutations in vemurafenib-resistant melanomas.^32^ Although frequently accompanied by atypical BRAF^G593S, L597R, K601E^ mutations, BRAF^V600E^ mutations or NRAS^Q61R^ mutations, MEK mutations are also found in primary melanomas in the absence of other driver mutations.^3^

Melanoma is a molecularly heterogeneous cancer with a mutation load that exceeds all other cancers.^5^ As a result, differentiating passenger from driver mutations or assessing their relative contribution is comparatively difficult. Several resistance mutations have even been proposed within the same patient or tumor, which substantially complicates the study of resistance mechanisms.^31^ Here, using a novel mouse model of melanoma that permits temporal regulation and targeted delivery of genes into somatic cells, we investigated whether MEK mutations, found in resistant melanomas, can drive the development and maintenance of melanoma *in vivo*.^33^ We also assessed their differential sensitivity to MEK or ERK inhibition and examined whether complete inhibition of mutant MEK would lead to sustained tumor regression or if resistance would develop.

## Results

### Relative activity of MEK mutants found in BRAF inhibitor-resistant melanoma

**Figure 2:**
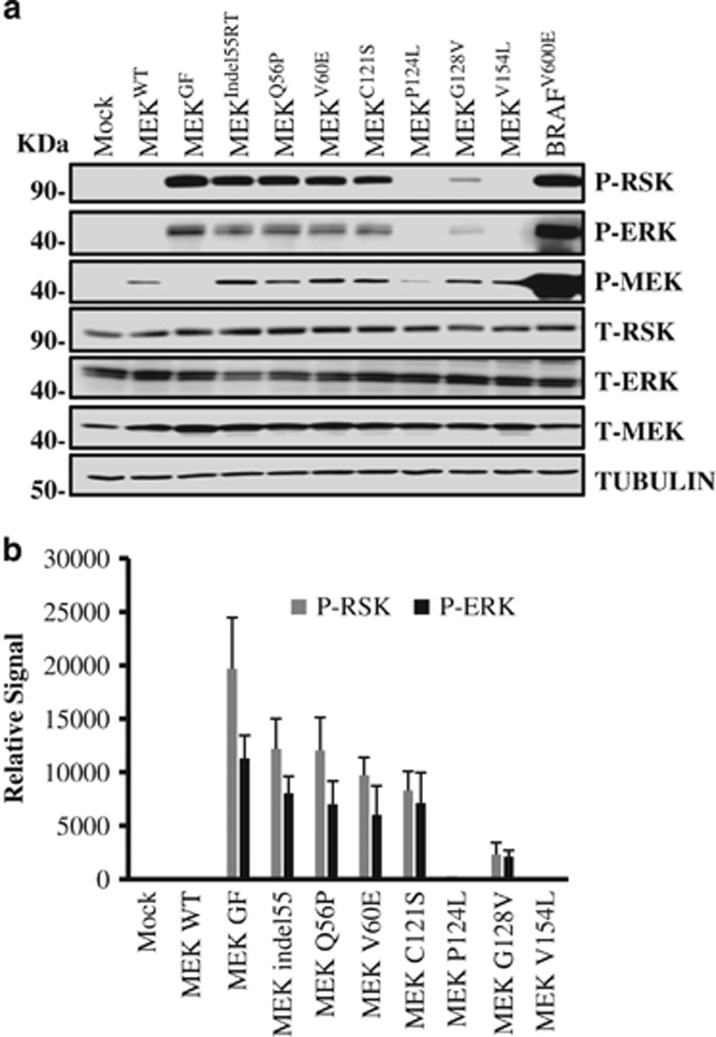
Comparative activity of MEK mutants found in BRAF^V600E^ inhibitor-resistant melanoma. (**a**) An immunoblot for MAPK pathway components in 293 FT cells transfected with plasmid DNA containing wild-type MEK, MEK^Indel55RT^, MEK^Q56P^, MEK^V60E^, MEK^C121S^, MEK^P124L^, MEK^G128V^, MEK^V154L^ and BRAF^V600E^ or empty vector controls. Both the transfections and immunoblotting were performed in triplicate. Tubulin was used as a loading control. (**b**) Densitometry measurements from three experimental replicates showing MEK^GF^ had higher activity than any of the naturally occurring mutants (*P*<0.05). Although MEK V60, Q56P and 55RT had similar activity, C121s trended toward lower activity, but this was not statistically significant. MEK^G128V^ had lower activity than C121S (*P*<0.05). P-ERK and P-RSK were not observed for P124L or V154L although P-MEK was detected.

Although several MEK mutations have been identified in resistant melanomas, whether these are passenger or driver mutations is not clear. To compare the activity of a panel of MEK1 mutations found in BRAF inhibitor-resistant melanoma cell lines, 293FT cells, which have low basal MAPK activity, were transfected with a bicistronic green fluorescent protein (GFP) vector containing either wild-type MEK, MEK^Indel55RT^, MEK^Q56P^, MEK^V60E^, MEK^C121S^, MEK^G128V^ MEK^P124L^, MEK^V154L^, MEK^GF^ or BRAF^V600E^. An empty GFP vector was transfected as an additional control. Following cell transfection, immunoblotting was used to evaluate the levels of phosphorylated ERK 1/2 (P-ERK), P-MEK and P-p90 ribosomal S6 kinase (P-RSK) at 48 h. Fluorescent microscopy demonstrated a 95% or higher transfection efficiency in transfected cells. These experiments were conducted in triplicate and then subjected to densitometry measurement. As expected, the BRAF^V600E^ positive control induced very high levels of P-ERK, and P-RSK, whereas wild-type MEK had no detectable activity. BRAF^V600E^ had 4.2 times the activity of MEK^GF^ (*P*<0.05 densitometry, data not shown for clarity). MEK^Indel55RT^, MEK^Q56P^, MEK^V60E^ and MEK^C121S^ all possessed similarly increased levels of (P-ERK/P-RSK) activity while MEK^G128V^ had significantly reduced activity (Figures 2a and b). Similar to other mutants found in resistant melanoma, MEK^P124L^ and MEK^V154L^ were both phosphorylated at Ser217 & Ser221; however, ERK and RSK phosphorylation was not detected. The phosphorylation-sites on MEK^GF^ are destroyed by the activating mutations, and consequently, no P-MEK band was detected.

### BRAF^V600E^ melanoma cell lines expressing MEK mutants are addicted to BRAF inhibitors

**Figure 3:**
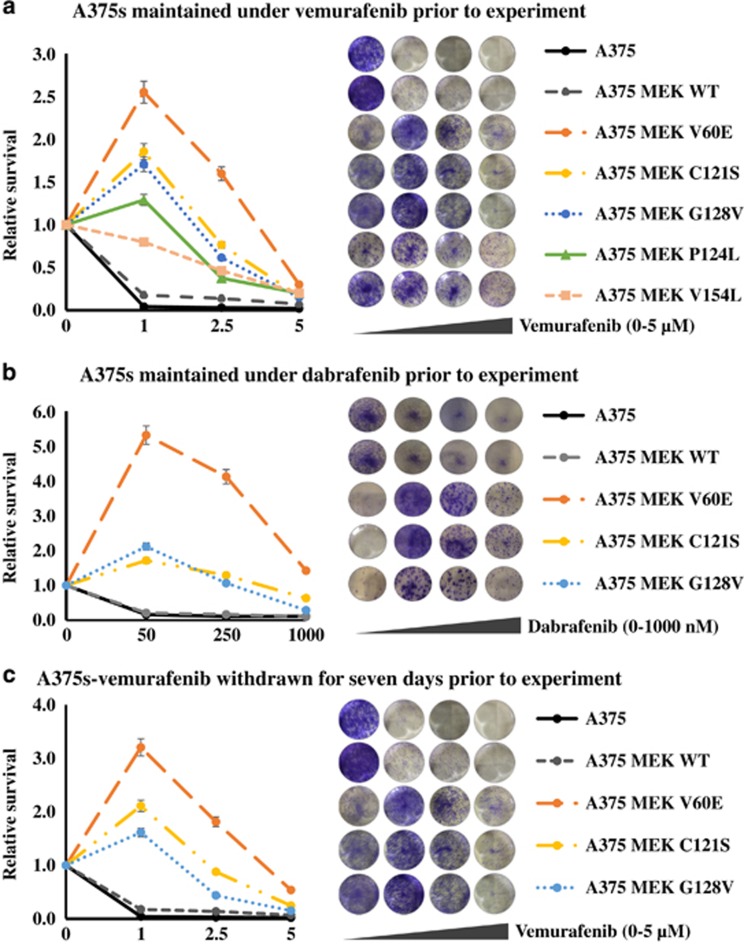
BRAF melanoma cells with MEK mutations are addicted to BRAF inhibition. (**a**) Melanoma cells infected with wild-type MEK, MEK^Indel55RT^, MEK^Q56P^, MEK^V60E^, MEK^C121S^, MEK^G128V^, MEK^P124L^, MEK^V154L^ or empty vector controls were maintained under 1 μm vemurafenib and treated with the indicated dosage of vemurafenib. (**b**) Cells were maintained under 50 nm dabrafenib and treated with the indicated dosage of dabrafenib. (**c**) Cells were maintained under 1 μm vemurafenib for 2 weeks followed by 7 days of withdrawal, then treated with the indicated dosage of vemurafenib. All experiments were performed in triplicate; representative wells at the escalating doses are shown to the left of the figure legend.

MEK mutants have been proposed to drive resistance to BRAF inhibition in melanoma. To assess resistance to BRAF, MEK and ERK inhibition, three well-characterized human BRAF^V600E^ melanoma cell lines (A375, M14 and SKMEL5) were infected with bicistronic GFP lentivirus containing either wild-type MEK, MEK^V60E^, MEK^C121S^, MEK^G128V^, MEK^P124L^ or MEK^V154L^ while they were maintained in media containing a BRAF inhibitor (vemurafenib or dabrafenib). Clonogenic assays, performed in triplicate, demonstrated that several of the MEK mutants not only confer resistance to BRAF inhibitors (vemurafenib or dabrafenib), but the cell lines become addicted to the presence of the inhibitors resulting in all exhibiting greater growth in the presence of the inhibitor than without, with the exception of WT and V154L MEK (Figure 3). This effect was even more pronounced in M14 and SKMEL5 cells (Supplementary Figure 1). The addiction was still evident even when the BRAF inhibitor was withdrawn for 7 days or when cells were maintained under 2.5-times the original dose of the inhibitor (Figure 3). We next used trametinib to assess the effect of MEK inhibition. In the dynamic inhibitor range used, with the exception of V154L, all MEK mutants assayed provided increased resistance to trametinib. However, this effect was abrogated by combining trametinib with either 1 μm vemurafenib or 50 nm dabrafenib. A similar, but much more pronounced effect, was observed using the ERK inhibitor, ulixertinib (Supplementary Figure 2). This suggests it is the increased level of ERK activation that the MEK mutants provide that requires additional inhibition.

**Figure 4:**
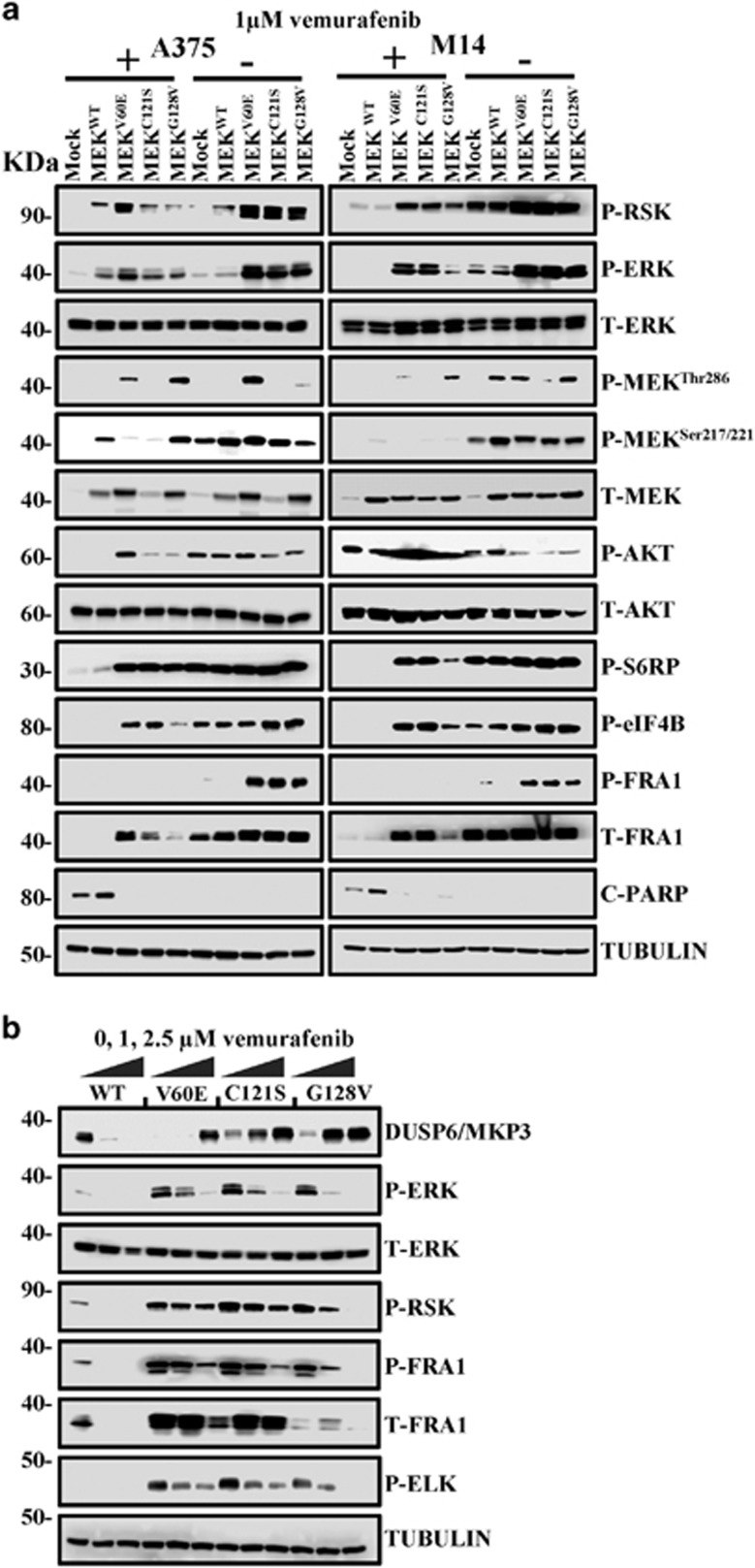
Withdrawal of BRAF inhibitors from BRAF inhibitor-resistant cells leads to enhanced MAPK signaling. (**a**) Melanoma cells expressing the indicated genes and maintained in 1 μm vemurafenib for 2 weeks were then either maintained for a further 72 h (+) or withdrawn (−) from vemurafenib. The expression and phosphorylation of MAPK family members (RSK, ERK and MEK), AKT, protein translation-related proteins S6 ribosomal protein (S6 RP), ELK, eukaryotic translation initiation factor 4B (eIF4B), apoptosis marker (cleaved poly ADP ribose polymerase) and Fos-related antigen 1 (FRA1) in response to drug withdrawal was detected by immunoblotting. (**b**) Melanoma cells treated with increasing (0, 1 and 2.5 μm) concentrations of vemurafenib. After 24 h, the expression and phosphorylation of ERK-related proteins including dual specificity phosphatase 6 (DUSP6) was detected by western blot.

Immunoblotting of extracts of MEK mutant cell lines in the presence or absence of the BRAF inhibitor, vemurafenib, demonstrated increased levels of phosphorylated and total FRA1, ELK, phosphorylated ERK and phosphorylated RSK in the MEK mutant cell lines compared with controls in the absence of BRAF inhibition. These levels were elevated even further when the drug was withdrawn (Figure 4a). Apoptosis was restricted to the control and MEK WT cells in the presence of BRAF inhibition as demonstrated by lack of poly ADP ribose polymerase cleavage. In contrast, protein translation-related proteins S6 RP, eIF4B and AKT were activated in all mutant cell lines in the presence or absence of BRAF inhibition. Phosphorylation of FRA1 by ERK increases protein stability and leads to overexpression of FRA1 (Entrez-Gene Id 8061) in cancer cells,^34^ and while increased FRA1 expression was observed in all mutant cell lines, poly ADP ribose polymerase was elevated in MEK mutant cell lines in the absence of BRAF inhibition. The increased levels of P-FRA1, P-ERK, P-RSK and decrease in cleaved poly ADP ribose polymerase was highly significant as shown by densitometry measurements taken from three independent experimental replicates (Supplementary Figure 3). These results were confirmed by demonstrating that withdrawal of BRAF inhibition in MEK mutant cell lines leads to increased MAPK activation possibly leading to negative feedback signaling in a manner proportional to the activity of the MEK mutant (Figure 4b). The downstream MAPK activity of the mutants was as expected directly related to resistance to BRAF inhibitors (V60E>C121S>G128V). No increase in apoptosis, autophagy or changes in cell cycle distribution was detected between MEK mutant cell lines following the withdrawal of vemurafenib as shown by flow cytometry assay results (Supplementary Figure 4). However, many more viable MEK mutant cells were observed in the absence of the inhibitor (Supplementary Figure 5A). These results suggest that the melanoma cell lines adapt to an optimal level of MAPK signaling and that concomitant activation of BRAF and MEK appears counterproductive.

### MEK activation promotes the transformation of melanocytes

Anchorage-independent growth is a hallmark of transformation. Accordingly, we used a soft agar assay to assess whether MEK mutants promote colony formation in melanocytes. Melanocytes isolated from the skin of *Dct::TVA;Cdkn2a*^*lox/lox*^*;Pten*^*lox/lox*^ mice were infected with an RCAS virus encoding Cre to delete *Cdkn2a* and *Pten.* Loss of Ink4a, Arf and Pten in these cells resulted in continuous proliferation without detectable senescence crisis in more than 20 population doublings, suggesting that they are immortal. These immortalized melanocytes were then infected with RCAS viruses containing GFP, BRaf^V600E^, wild-type MEK or MEK^GF^. Immunoblotting of cell lysates confirmed expression in the infected melanocytes. To ensure that MEK^GF^ and BRaf^V600E^ were active, we evaluated the levels of phosphorylated ERK 1/2 (P-ERK) following serum starvation. MEK^GF^ and BRaf^V600E^ induced elevated levels of P-ERK, whereas GFP and wild-type MEK controls did not (Supplementary Figure 5B). To assess the ability of MEK^GF^ and BRaf^V600E^ to induce anchorage-independent growth *in vitro*, a soft agar colony growth assay was performed. Whereas GFP- and MEK^WT^-expressing immortalized melanocytes were unable to grow in soft agar, MEK^GF^ and BRaf^V600E^-expressing immortalized melanocytes formed numerous colonies, demonstrating their ability to grow in an anchorage-independent manner (Supplementary Figure 5C). These clonal cell populations were highly pigmented, confirming their melanocytic origin.

### MEK activation cooperates with *Cdkn2a* and *Pten* inactivation to induce melanoma

**Figure 5:**
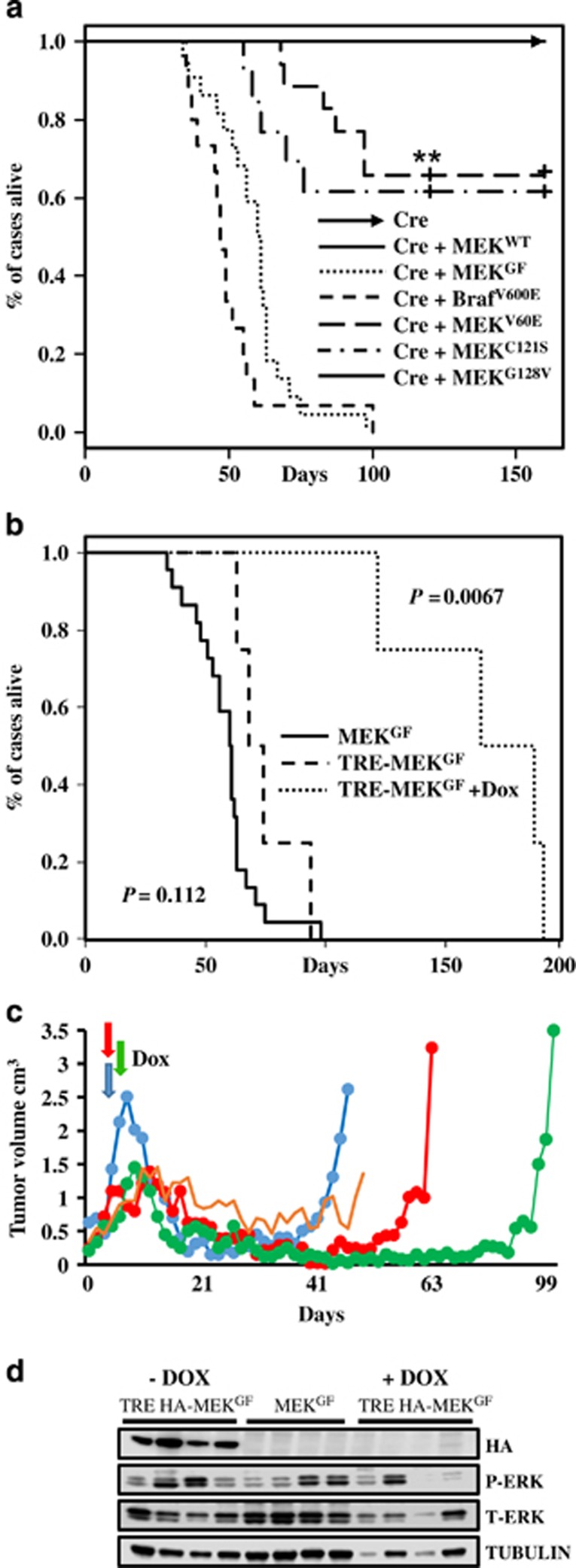
MEK cooperates with Cdkn2a and Pten loss in the development of melanomas *in vivo.* (**a**) Kaplan–Meier percent survival curves for BRAF and MEK tumors. *Dct::TVA; Cdkn2a*^*lox/lox*^*; Pten*^*lox/lox*^ mice were injected with viruses encoding Cre (raised arrow headed line, *n*=17 tumor incidence 0/17) or wild-type MEK+Cre (solid line, *n*=12, tumor incidence 0/12) or BRAF^V600E^+Cre (closely dashed line, *n*=15, incidence 15/15) or MEK^GF^+Cre (dotted line tumor incidence 22/22). Mice were also injected with MEK^V60E^+Cre (wide dashed line, *n*=18, incidence 6/18) or MEK^C121S^+Cre (dotted and dashed line, *n*=13, tumor incidence 5/13) or MEK^G128V^+Cre (solid line, *n*=7 tumor incidence 0/7). At the time of publication 7 MEK^V60E^ & Cre and 4 MEK^C121S^+Cre mice remain tumor free at just over 120 days of age and 5 MEK^V60E^ & Cre and 4 MEK^C121S^ mice remained tumor free until the experiment end point of 160 days. No significant difference was observed between MEK^C121S^ and MEK^V60E^ *P*=0.563. A significant difference was observed between BRAF^V600E^ & Cre and MEK^GF^+Cre (*P*=0.0401). A significant increase in survival is evident between MEK^GF^ and MEK^V60E^ (*P*=3.19^−09^), and MEK^GF^ and MEK^C121S^ (*P*=4.87^−05^). (**b**) Kaplan–Meier survival curves demonstrating the effect of genetic MEK inhibition. *Dct::TVA;Cdkn2a*^*lox/lox*^*;Pten*^*lox/lox*^ mice were injected with viruses encoding Tet-off P2A Cre+TRE-MEK^GF^. Mice were monitored for tumor formation and randomized to receive a Dox diet or control diet when tumors were measured at 1.0 cm^3^ (Dox diet dotted line, *n*=4, control diet dashed line, *n*=4). A significant increase in survival was found between Dox-treated and control mice (*P*=0.0067). No difference in survival was observed between the Tet-off P2A Cre+TRE-MEK^GF^ control mice and MEK^GF^+Cre tumors shown again in this panel for comparison (solid line, *P*=0.112). (**c**) MEK inhibition leads to tumor regression and recurrence. Plot showing the volume of four Tet-off P2A Cre+TRE-MEK^GF^ tumors from the first tumor measurement until death. Mice were treated with Dox when tumors were measured at 1.0 cm^3^ (denoted by colored arrows) and killed when tumors reached 2.5 cm^3^; the oldest mouse was killed at 192 days. One mouse developed stable disease before being found dead. (**d**) Virally delivered MEK^GF^ expression was detected in proteins extracted from mouse melanomas using an antibody for the HA epitope tag in tumors induced with RCAN TRE-HA-MEK^GF^+RCAS Tet-off P2A Cre but absent from the recurring Dox-treated tumors and absent from untagged RCAS MEK^GF^ and Cre control tumors. MAPK activity was evaluated by blotting for phosphorylated and total ERK 1/2.

To study the role of MEK mutants in melanoma development, resistance and maintenance, we utilized the RCAS/TVA melanoma mouse model to validate MEK oncogenic mutations *in vivo*.^33, 35^ Using this system, multiple genetic alterations can be introduced into the same cell, in the context of an unaltered microenvironment. Newborn Dct:*:TVA;Cdkn2a*^*lox/lox*^*;Pten*^*lox/lox*^ mice were injected subcutaneously with either RCAS Cre, MEK^GF^+Cre, MEK^WT^+Cre, MEK^V60E^+Cre, MEK^C121S^+Cre or MEK^G128V^+Cre virus. For comparative purposes, we also delivered BRaf^V600E^+Cre. Mice were subsequently monitored for tumor growth and development. All of the mice injected with MEK^GF^+Cre developed melanomas (22/22) and the mean survival was 58 days (range 34–98; Figure 5a). Thirty three percent (6/18) of the mice injected with MEK^V60E^+Cre developed tumors within 120 days, with the mean survival of tumor-bearing mice at 83.5 days (range 68–97 days). Forty percent (5/13) of mice injected with MEK^C121S^+Cre developed tumors and the mean survival of tumor-bearing mice in this cohort was 64 days (range 55–70 days). At the time of publication, 7 MEK^V60E^+Cre and 4 MEK^C121S^+Cre mice remained tumor free at just over 120 days, and 5 MEK^V60E^ & Cre and 4 MEK^C121S^ mice remained tumor free until 160 days of age. No tumors developed in the mice injected with Cre+MEK^G128V^ within 160 days (0/7). All of the mice injected with BRaf^V600E^ (15/15) developed melanoma within 120 days, and the mean survival was 50 days (range 36–100 days; Figure 5a). No significant difference was observed between MEK^C121S^+Cre and MEK^V60E^+Cre (*P*=0.563). The difference in survival between MEK^GF^+Cre and BRaf^V600E^+Cre tumor-bearing mice just reached statistical significance (*P*=0.040). However, the large survival variances for MEK^GF^ and BRaf^V600E^ were driven by a single slow growing tumor in each cohort. If these outliers are removed, variance drops significantly (MEK^GF^ mean 56.5, range 34–75 vs BRaf^V600E^ mean 46.5, range 36–58 days) and the statistical difference between the two cohorts becomes highly significant (*P*=2.04^−04^). The differences between MEK^GF^ and MEK^V60E^ (*P*=3.19^−09^) or MEK^C121S^ (*P*=4.87^−05^) were highly significant with the MEK^GF^ cohort demonstrating reduced tumor latency. All TVA-negative controls (*n*=36) and controls infected with MEK^WT^+Cre (*n*=12) or Cre alone (*n*=17) remained tumor free. Lung metastases were detected in 5% (1/22) of MEK^GF^ tumor-bearing mice by a board certified pathologist (AHG). No other tumor types or brain metastases were detected. No significant difference was observed between the incidence or survival of BRaf^V600E^+Cre tumors in the *Dct::TVA;Cdkn2a*^*lox/lox*^*;Pten*^*lox/lox*^ mice reported here and those induced with Cre alone in *Dct::TVA;BRaf*^*CA*^*;Cdkn2a*^*lox/lox*^*;Pten*^*lox/lox*^ mice we have reported previously (*P*=0.145).^35^ The expression of Cre, MEK^GF^ and BRaf^V600E^ was confirmed using RT-PCR on RNA extracted from melanoma tissue. Cre activity was confirmed by PCR for the *Pten* exon 5 deletion. Western blotting of protein lysates from Braf^V600E^ tumors confirmed the expression of the mutant protein (Supplementary Figures 6a and b).

### Suppression of MEK^GF^ results in prolonged tumor regression but all tumors recur

As discussed earlier, multiple animal and human studies have assessed the efficacy of selumetinib, trametinib and cobimetinib alone or in combination with BRAF inhibitors. In culture, these inhibitors are highly effective and at the right dose completely block MAPK signaling.^17, 31, 36^ Despite this, combinatorial MEK and BRAF inhibition has not proven to be curative in most patients and tumors nearly always recur.^12, 13^ We reasoned the crux of the problem might not lie with the inhibitors themselves, but the inability to specifically and effectivity target tumor cells in a prolonged manner at a dose that does not have disproportionate effects on processes required by normal tissues.^37, 38^ To test this hypothesis, we used a genetic approach to examine the effects of complete inhibition of mutant MEK on melanoma maintenance *in vivo*. To allow for regulation of MEK^GF^ expression *in vivo* using the Tet-regulated system, we utilized the RCAN(A) vector as opposed to RCAS, wherein expression is decoupled from the viral long-terminal repeat through the deletion of a key splice acceptor site,^39^ and is instead driven from a Tet-responsive element (TRE). Expression from the TRE requires the presence of a tetracycline transcriptional activator such as Tet-off. In the context of Tet-off, the Tet-responsive MEK is repressed in the presence of doxycycline (Dox). Expression and activity of human influenza hemagglutinin (HA) epitope tagged MEK^GF^ was first validated *in vitro* in the context of Tet-off±Dox before the *in vivo* experiments (Supplementary Figure 4D). Newborn *Dct::TVA;Cdkn2a*^*lox/lox*^*;Pten*^*lox/lox*^mice were injected subcutaneously with the RCAS Tet-off P2A Cre and RCAN TRE-HA-MEK^GF^ virus. Tumor development and growth were monitored daily. When the tumors reached 1000 mm^3^, the mice were randomly assigned to receive standard feed (untreated) or Dox-containing food to suppress MEK expression and determine whether down-regulation of MEK expression results in tumor regression. Survival rates were compared between all cohorts of untreated mice using a log-rank test of the Kaplan–Meier estimate of survival (Figure 5b). No difference in the survival of tumor-bearing mice was observed between RCAS MEK^GF^ (*n*=22) and RCAN TRE HA-MEK^GF^+Tet-off cohorts (*n*=4; *P*=0.112). The administration of Dox and subsequent loss of MEK expression significantly increased survival (*P*=0.0067). No tumors developed in a cohort of 11 control mice injected with RCAS Tet-off P2A Cre alone throughout the entire experimental period. The mean survival for the untreated RCAN TRE HA-MEK^GF^+Tet-off mice was 74 days (range 63–98 days) and the mean survival for the Dox-treated mice (*n*=4) was 166 days (range 122–191 days). One animal experienced a period of stable disease, but all other mice experienced near complete tumor regression (Figure 5c). However, following a prolonged latency, all tumors eventually became resistant to MEK inhibition. To rule out loss of Tet-regulation of MEK as a mechanism(s) of resistance responsible for mediating tumor recurrence, we evaluated all tumor tissue for expression of virally delivered MEK by immunoblotting and immunohistochemistry (IHC) for the HA epitope tag on MEK (Figure 5d). HA expression was absent from Dox-treated tumors but present in untreated controls. Further assessment revealed that several of the resistant tumors appeared to have reactivated the MAPK pathway through an alternate mechanism (Figure 5d).

### Tumor histology

**Figure 6:**
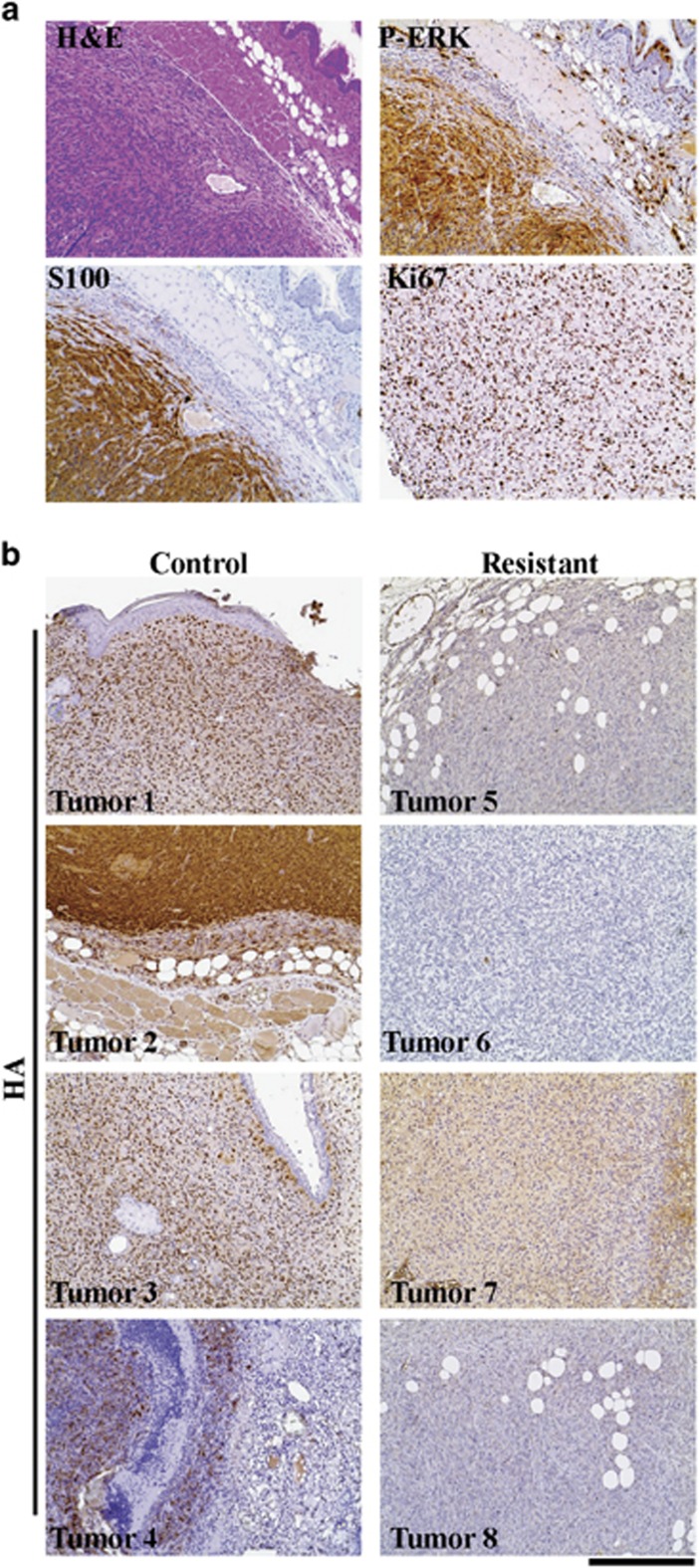
Melanoma histology. (**a**) The immunoprofile of representative MEK^GF^ melanoma. MEK melanomas were highly vascular and consisted primarily of short spindle cells exhibiting high-grade nuclear features and prominent nucleoli. They invaded into subcutaneous fat, muscle and cartilage. IHC for S100 demonstrated the melanocytic origin of the tumors. IHC for P-ERK demonstrated canonical MAPK pathway activation. Assessment of cellular proliferation was performed on slides with uniform tumor cellularity using IHC for the cellular proliferation marker Ki67 and demonstrated that all of the tumors were highly proliferative. IHC sections were counterstained with hematoxylin. A hematoxylin and eosin (H&E) stained tumor section is provided for comparison. (**b**) *In situ* assessment of MEK expression in Dox resistant tumors. Resistant tumor sections and controls were assessed by IHC for expression of the HA epitope tag on virally delivered MEK^GF^. All control tumors showed a mixture of nuclear or cytoplasmic HA expression that was not detected in resistant tumors, which demonstrated continued TET-regulated suppression of MEK^GF^ expression with Dox. IHC sections were counterstained with hematoxylin. The scale bar represents 200 μm.

The MEK and BRaf^V600E^ melanomas were indistinguishable from each other and from the melanomas arising in *Dct::TVA;Braf*^*CA*^*;Cdkn2a*^*lox/lox*^*;Pten*^*lox/lox*^ mice^36^ infected with Cre, as we have previously reported.^35^ As with *Dct::TVA* NRas^Q61R^ ^33^ and BRaf^CA^^ 4^ tumors, all of the melanomas arising in this study were highly invasive and consisted primarily of short spindle cells exhibiting high-grade nuclear features and prominent nucleoli (Figure 6a). The melanocytic origin of tumors arising in both the *Dct::TVA* mouse models has previously been established using IHC for S100, HMB-45 and MART-1.^33^ The melanocytic origin of the MEK tumor cohorts was again confirmed by immunostaining for S100. Staining for P-ERK revealed consistently high levels of canonical MAPK activation in all tumors. All tumors had similar immuno-profiles, with a slight variation in the expression of individual markers. IHC for the cellular proliferation marker, Ki67, demonstrated that all tumors were highly proliferative (Figure 6a). RCAN TRE HA-MEK^GF^+Tet-off tumor sections were also evaluated for expression of virally delivered MEK using immunostaining for the HA epitope tag. Although both nuclear and cytoplasmic HA expression was present in untreated tumors, HA expression was entirely absent from Dox-treated tumors (Figure 6b). Immunostaining, using antibodies to detect cleaved caspase 3 demonstrated increased apoptosis in an TRE HA-MEK^GF^+Tet-off tumor collected following 96 h of Dox treatment compared to an untreated TRE HA-MEK^GF^+Tet-off tumor (Supplementary Figure 7). Large areas of dead tissue were also apparent in the Dox-treated tumor, which had lost a third of its volume in the preceding 96 h.

## Discussion

Here, we report for the first time that melanoma initiation, growth and maintenance can be driven by activating MEK mutations. This is significant because a wide range of MEK mutations are frequently found in vemurafenib/dabrafenib-resistant melanomas and in primary untreated melanomas.^3^ That MEK has shown susceptibility to a particularly broad spectrum of gain-of-function mutations highlights the many escape routes to activation and its evolutionary flexibility in the context of melanoma pathogenesis. Using the RCAS/Dct:TVA mouse model of melanoma, we found that gain-of-function MEK1 mutations (V60E, C121S and GF) are capable of transforming melanoma cells *in vitro* and driving high-grade melanoma in the context of *Cdkn2a* and *Pten* loss. The ability of any particular gain-of-function MEK mutation to drive melanoma is directly related to its ability to activate MAPK effectors, most immediately ERK. Not all MEK mutations found in melanomas treated with MEK and BRAF^V600E^ inhibitors will be activating. In fact, some we have found (for example, MEK^V154^) are largely passenger mutations conferring little to no growth advantage whereas others (for example, V60E, C121S and G128V) confer not only a growth advantage, but increased insensitivity to BRAF and/or MEK inhibitors or primarily act to confer resistance to MEK inhibitors (for example, MEK^P124L^). Our results further show that activating MEK mutations that have arisen in melanoma cells with BRAF^V600E^ mutations result in suboptimal melanoma growth following the withdrawal of BRAF inhibition. This suggests that melanoma cells have selected through clonal evolution for an optimal level of MAPK signaling, and that the additive effects of a MEK mutation and a BRAF mutation are counterproductive. This effect was clearly still evident several weeks after BRAF inhibitor withdrawal. In this manner, that BRAF cell lines with activating MEK mutations are ‘addicted’ to BRAF inhibitors is apparent. Interestingly, we found that the MEK mutations we studied provided additional resistance to both MEK and ERK inhibitors in a manner clearly related to their ability to activate ERK because the effect was more pronounced for ERK inhibition than it was for MEK inhibition. This dichotomy suggests that some MEK mutants are not, as has been widely reported^11, 30, 40^ providing direct resistance to the MEK inhibitor. Instead, it is the increased level of ERK activation, which they provide, that requires additional inhibition. The increased resistance to ERK inhibition by MEK mutants, which we report herein, is highly novel and has not been previously reported. Counter intuitively, because of the clear addition of the MEK mutant cell lines to BRAF inhibition, exposure of MEK mutant cell lines to both BRAF and MEK inhibitors gave the best response, which is a clinically relevant finding. Narita Y *et al.* provide an example of how MEK mutants (MEK^C121S^) can respond very differently to the alternative MEK inhibitors, E6201 and selumetinib. However, we have shown that MEK^C121S^ signaling can be blocked by a moderate increase in the dose of trametinib, a dose that remains significantly lower than that of selumetinib or E6201.^40^ MEK mutations may be less common than BRAF^V600E^ mutations in primary melanomas, because in our cellular assay, their relative ability to stimulate the MAPK pathway was less than a quarter of the ERK activating activity of BRAF^V600E^. Furthermore, the speed of tumor formation in our mouse model was proportionally related to the apparent MAPK activity of the mutants.

Although very effective MEK inhibitors exist, complete and sustained systemic MEK inhibition is probably neither desirable nor obtainable in melanoma patients due to the importance of the MAPK pathway to normal cells. Accordingly, clinical trials using MEK inhibitors alone have been far less successful than for BRAF^V600E^ inhibitors in BRAF^V600E^ melanoma.^41, 42^ By using a genetic approach, we were able to circumvent these issues as a means of precisely determining if inactivation of MEK could be an effective treatment for melanoma. Tet-responsive MEK mutant melanomas treated with Dox regressed significantly and showed increased survival compared with untreated controls; however, after a long latency, all tumors subsequently developed resistance. This result does not infer that MEK is an inappropriate therapeutic target; to the contrary, it infers that as with BRAF mutated melanomas treated with BRAF inhibitors, a subpopulation of melanoma cells survive and lie dormant prior to reoccurrence. These results also infer new and improved MEK or BRAF inhibitors are likely not the solution. In this manner, our melanoma mouse model is ideal for the study of tumor dormancy and resistance, both to BRAF and MEK inhibition, and is well suited to determine and validate additional genetic alterations found in recurrent tumors that may be responsible for resistance and regrowth.

Our results provide strong evidence showing that a broad spectrum of MEK mutations over-stimulate the MAPK pathway and ultimately generate tumors that mimic the tumor pathology and aggressive nature of BRAF^V600E^-driven melanomas. Our findings are clinically relevant for several reasons. First, we have shown that an activating MEK mutation is not simply a benign passenger mutation but, instead, a potent and nocuous mutation that promotes cell transformation and aggressive malignancy. Owing to the propensity of MEK to evolve an array of activating mutations, our results support the co-targeting of alternative MAPK pathway members in conjunction with BRAF inhibitors for patients with high-grade, BRAF^V600E^-driven melanoma. Given that mutations that reactivate the MAPK pathway are relatively common in BRAF inhibitor-resistant melanomas and that melanomas evolve to an optimal level of MAPK signaling, escalating inhibitor doses over time from the lowest active to the highest tolerated, and the eventual addition of ERK inhibition and treatment holidays may improve patient outcomes. Whether or not MEK gain-of-function mutations are present pre- or post-BRAF and/or MEK inhibition (that is, a driver or contributor to oncogenesis versus an evolved resistance mechanism) are factors that will require careful consideration and clinical planning when developing a treatment strategy for melanoma patients. What is almost certain is that like BRAF-activating mutations, MEK-activating mutations combined with loss of tumor suppressors in melanocytes are likely to produce melanoma and may require special considerations in the clinic to optimize patient care.

## Materials and methods

### Animal model

*Dct::TVA;Cdkn2a*^*lox/lox*^; mice were crossed with mice carrying a floxed Pten allele (a gift from M. McMahon and M. Bosenberg) to generate Dct*::TVA;Cdkn2a*^*lox/lox*^*;Pten*^*lox/lox*^ mice. All mice were maintained on a mixed C57Bl/6 and FVB/N background by random interbreeding. Litters of newborn male and female mice were infected as previously described.^33^ The number of mice was determined based on previous experience with this model to obtain statistically meaningful results at the 95% confidence interval. Sample sizes were as follow RCAS Cre (*n*=17), MEK^GF^+Cre (*n*=22), MEK^WT^+Cre (*n*=12), MEK^V60E^+Cre (*n*=18), MEK^C121S^+Cre (*n*=13), or MEK^G128V^+Cre (*n*=7), Tet-off P2A Cre (*n*=11) and Tet-off P2A Cre+TRE-MEK^GF^ (*n*=8), and BRaf^V600E^+Cre (*n*=15). DNA was prepared from tail biopsies and genotyped at 17 days as described.^33, 35^ Mating pairs were randomized with respect to viral infection of their litters. No blinding approach was used during this study. No infected mouse with the correct genotype was excluded from analysis. Censored survival data was analyzed using a log-rank test of the Kaplan–Meier estimate of survival. To compare means, two-tailed Student’s *t*-test was used. *P*-values below 0.05 were considered significant. All animal experiments were performed in compliance with ‘Care and Use of Animals’ in Association for Assessment and Accreditation of Laboratory Animal Care (AAALAC)-accredited facilities, and approved by the University of Minnesota IACUC.

### Vectors

The retroviral vectors used in this study were replication-competent avian leukosis virus long-terminal repeat, splice acceptor and bryan polymerase-containing vectors of envelope subgroup A, designated RCASBP(A) and replication-competent, avian leukosis long-terminal repeat, no splice acceptor designated RCANBP(A). The RCAS(A) receptor is encoded by the Tumor Viruses A (TVA) gene that is normally expressed in avian cells. In transgenic mice express the TVA receptor under the control of the dopachrome tautomerase (DCT) promoter this allows the targeting of the virus specifically to melanocytes *in vivo*. In mammalian cells that express TVA, the viral vector is capable of stably integrating into the DNA and expressing the inserted experimental gene, but the virus is replication-defective. RCASBP(A) Cre, Tet-off and MEK^GF^ have been previously described,^37, 43^ as has RCANBP(A)TRE.^44^ MEK^GF^ is a constitutively active MEK1 with S218E and S222D substitutions and lacking residues 32–51 (ΔN3). MEK mutants were created using PCR mutagenesis and cloned into pCR8/GW/TOPO (Invitrogen, Carlsbad, CA, USA) and verified by Sanger sequencing. Genes were then recombined into the RCAS, RCAN or lentiviral vectors (FG12-cmv-DV-Ubic-GFP) using LR Clonase (Invitrogen) for subsequent transfection and or viral production, infection, and stable expression.^45^ Primer sequences and cloning strategies are available on request. ‘BRaf^V600E^’ used for these studies was cloned from a mouse melanocyte complementary DNA library using primers for *Braf* transcript (ENSMUST000000024870). Human BRAF^V600E^ has been previously described.^46^ Viral propagation, and *in vivo* and *in vitro* infection was performed as previously described.^33^

### Cell culture

Melanoma cell lines from the American Type Culture Collection (ATCC- A375s, SKMEL5s) or National Cancer Institute (M14s) were grown in Roswell Park Memorial Institute medium supplemented with 5% fetal bovine serum (FBS). DF-1 cells (ATCC) were grown in Dulbecco's Modified Eagle Medium-high glucose media supplemented with 10% FBS (Invitrogen), 2.5 ml gentamicin, and maintained at 39 °C. Mouse melanocytes were grown in 254 media supplemented with 10% FBS, HMGS, 2.5 ml gentamicin and maintained at 37 °C. 293FTs (ATCC) were maintained in Dulbecco's Modified Eagle Medium supplemented with 5% FBS. Transfection of FG12 vector DNA into 293FTs, 0.5 μg of DNA per 200 K cells, was performed using Lipofectamine 2000 (Invitrogen) in the absence of serum according to the manufacturer’s instructions. Assessment of anchorage-independent growth was performed as described.^46^ Clonogenic assay: melanoma cells were seeded at 3000 cells/well in 6-well plates. Cells were stained with 0.25% crystal violet Crystal Violet (C0775, Sigma-Aldrich, St. Louis, MO, USA) in 50% methanol. After drying, the crystal violate was dissolved in 10% acetic acid and optical density measured at 450 nm (Epoch microplate reader, Biotek, Winooski, VT, USA). Drugs: Vemurafenib, dabrafenib, ulixertinb and trametinib were purchased from Selleckchem, (Houston, TX, USA). All experiments were performed using biological replicates in triplicate.

### Western blotting

Immunostaining for HA was performed using an anti-HA monoclonal antibody (HA.11, Covance, Berkeley, CA, USA) at a 1:1000 dilution, and for V5 using a mouse monoclonal antibody targeting V5 (Sigma, St. Louis, MO, USA) at a 1:1000 dilution. Detection of BRAF^V600E^ was performed using a 1:1000 dilution of the anti-V600E antibody RM8 (RevMAb Biosciences, San Francisco, CA, USA). Tubulin was detected using a tubulin HRP antibody diluted 1:5000 (21058 Abcam, MA, USA). Detection of MAPK activation was performed using a 1:2000 dilution of a rabbit monoclonal antibody directed against phosphorylation of ERK at Thr202 and Tyr204, and phospho-p90RSK at Ser380 (4370 & 11989, Cell Signaling, Boston, MA, USA). The following Cell Signaling antibodies were also used at 1:1000: anti-ERK (9102), P-AKT (4060), T-AKT (4691), P-S6 RP (4858), P-eIF4B (3591), P-FRA1 (5841), cleaved poly ADP ribose polymerase (5625), T-MEK1 (9146), T-RSK (14813), P-MEK Ser217/221 (9154), P-MEK Thr286 (9127), DUSP6/MKP3 (3058) and P-Elk (9181). For mouse on mouse tissue samples a conformation specific secondary rat anti-mouse IgG-HRP (ab131368 Abcam) antibody diluted 1:1000 was used. All other blots were incubated with an anti-mouse or rabbit IgG-HRP secondary antibody diluted (7074, 7076 Cell Signaling) 1:2000 for 1 h at room temperature. Blots were then incubated with premium sure ECL (Li-Cor, Lincoln, NE, USA) and imaged using the Li-Cor C*-*DiGit chemiluminescence imager. Western blot density analyses were normalized against internal loading controls and performed on biological replicates in triplicate using Li-Cor Image studio digits software. To compare means, two-tailed Student’s *t*-test was used. *P*-values below 0.05 were considered significant.

### Immunohistochemistry

Analysis of S100 expression was performed using a rabbit polyclonal antibody Z0331 (1:400) for S100 (Dako; Glostrup, Denmark). Detection of MAPK activation was performed using a 1:100 dilution of an antibody against phospho-ERK (4370, Cell Signaling). Cell proliferation was detected using a 1:250 dilution of a rabbit monoclonal antibody against Ki67 (RM-9106-R7 Thermo Scientific, Waltham, MA, USA). IHC HA was performed using a 1:200) dilution of the HA.11 antibody. Apoptosis detection was performed using a 1:250 dilution of the cleaved caspase-3 antibody (9579 Cell Signaling). Controls were conducted without primary antibody on corresponding sections. Detection of HRP activity was performed using DAB (Cell Signaling). Sections were counterstained with hematoxylin.

### RT-PCR

Total RNA & DNA was extracted from mouse primary tumors using TRIzol reagent (Invitrogen) and chloroform. The SuperScript IV First-Strand Synthesis system (Invitrogen) was used to synthesize complementary DNA from DNase-treated RNA. PCR reactions were performed using an AccuStart II Mouse genotyping kit (Quanta biosciences, Gaithersburg, MD, USA). The primers used and amplicon sizes are available on request.
