# Supplementary figures and images for: Activated MEK cooperates with Cdkn2a and Pten loss to promote the development and maintenance of melanoma
Source: Oncogene. 2017 Mar 6;36(27):3842–51. doi: 10.1038/onc.2016.526 (PMC5501768; doi:10.1038/onc.2016.526)

# Supplementary Figure 1.

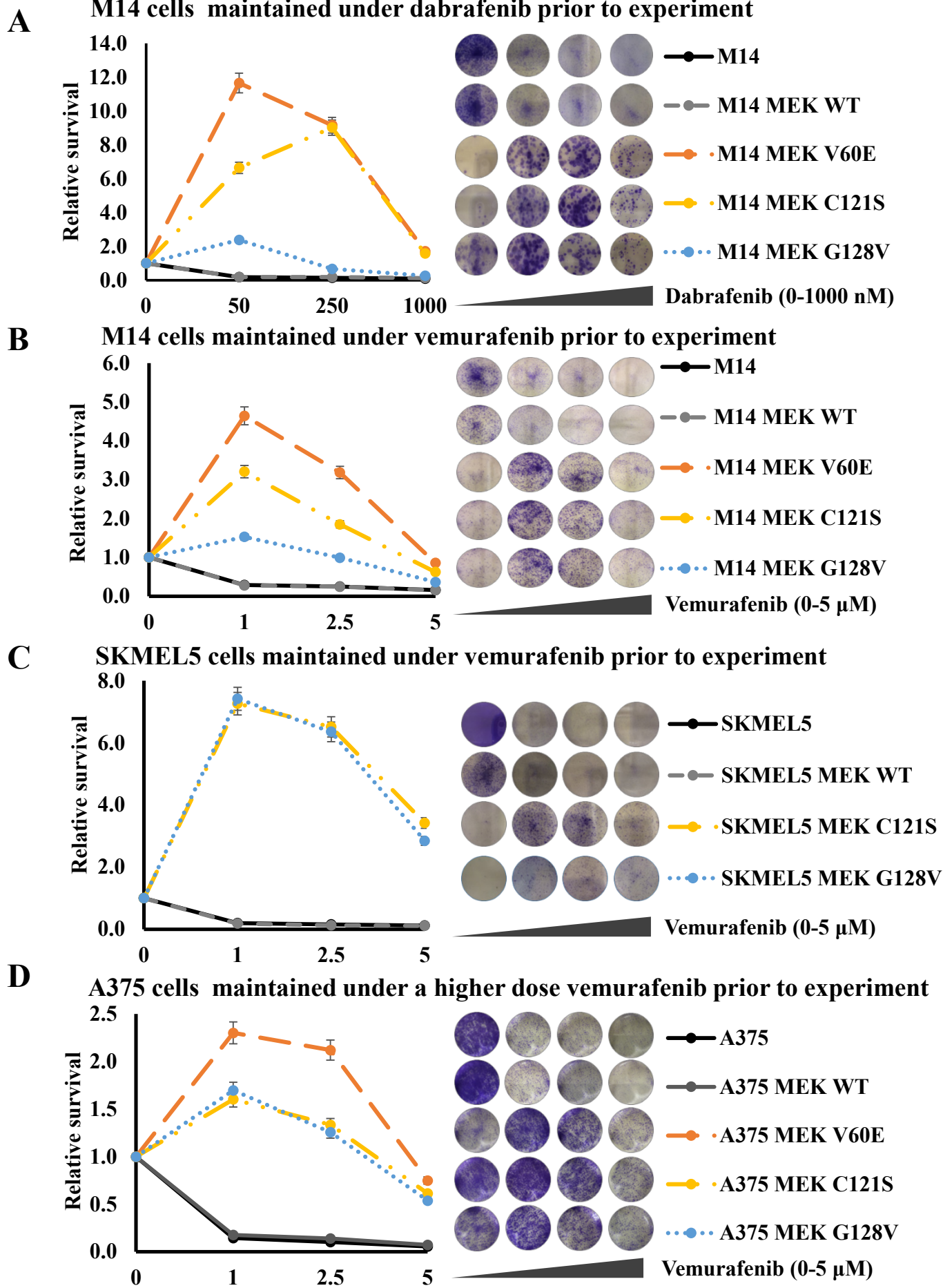

Supplement: Supplementary Figure 1 [file onc2016526x1.pdf]

# Supplementary Figure 2.

A

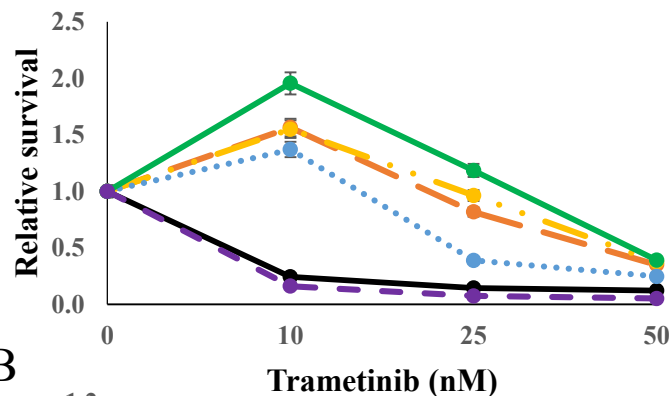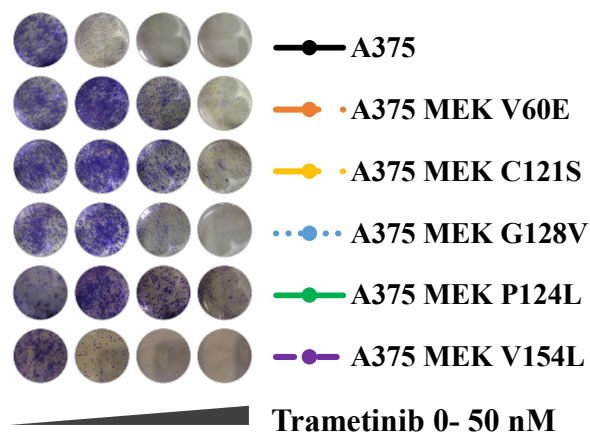

B

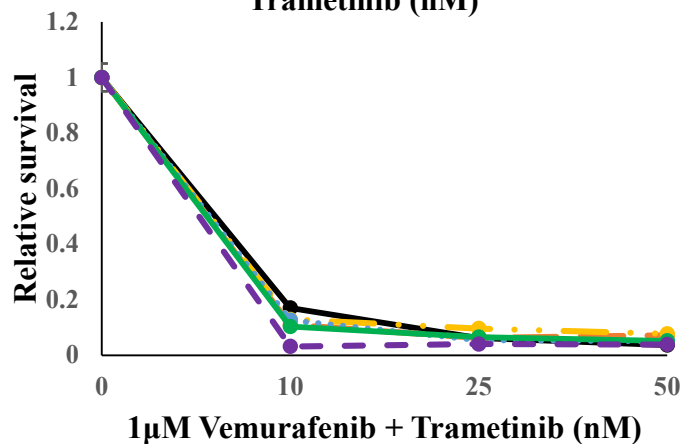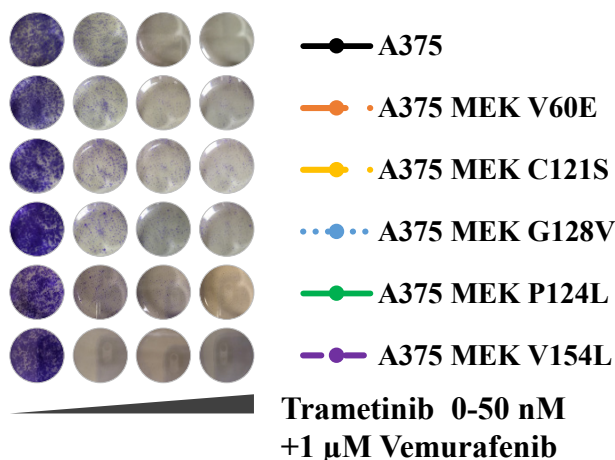

C

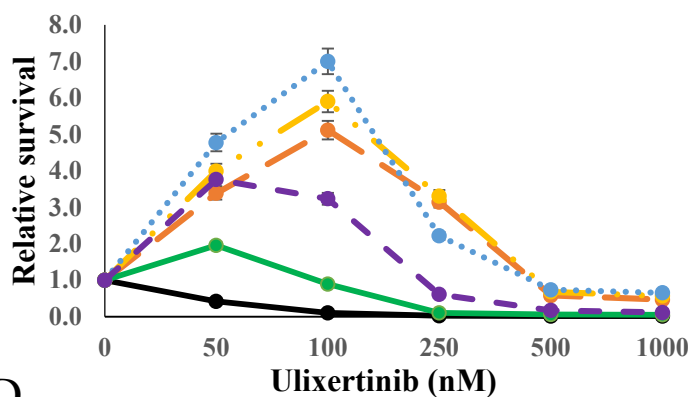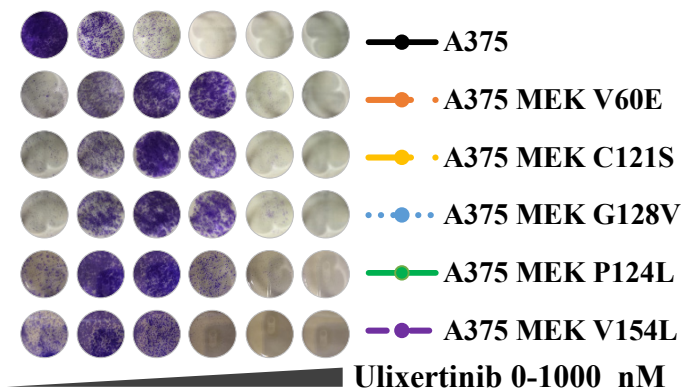

D

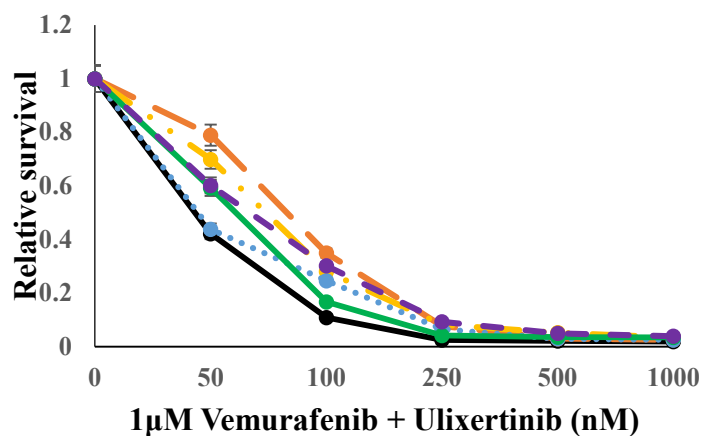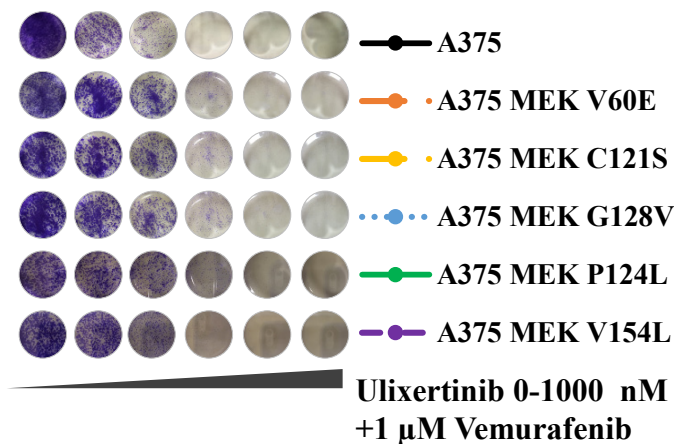

Supplement: Supplementary Figure 2 [file onc2016526x2.pdf]

# Supplementary Figure 3.

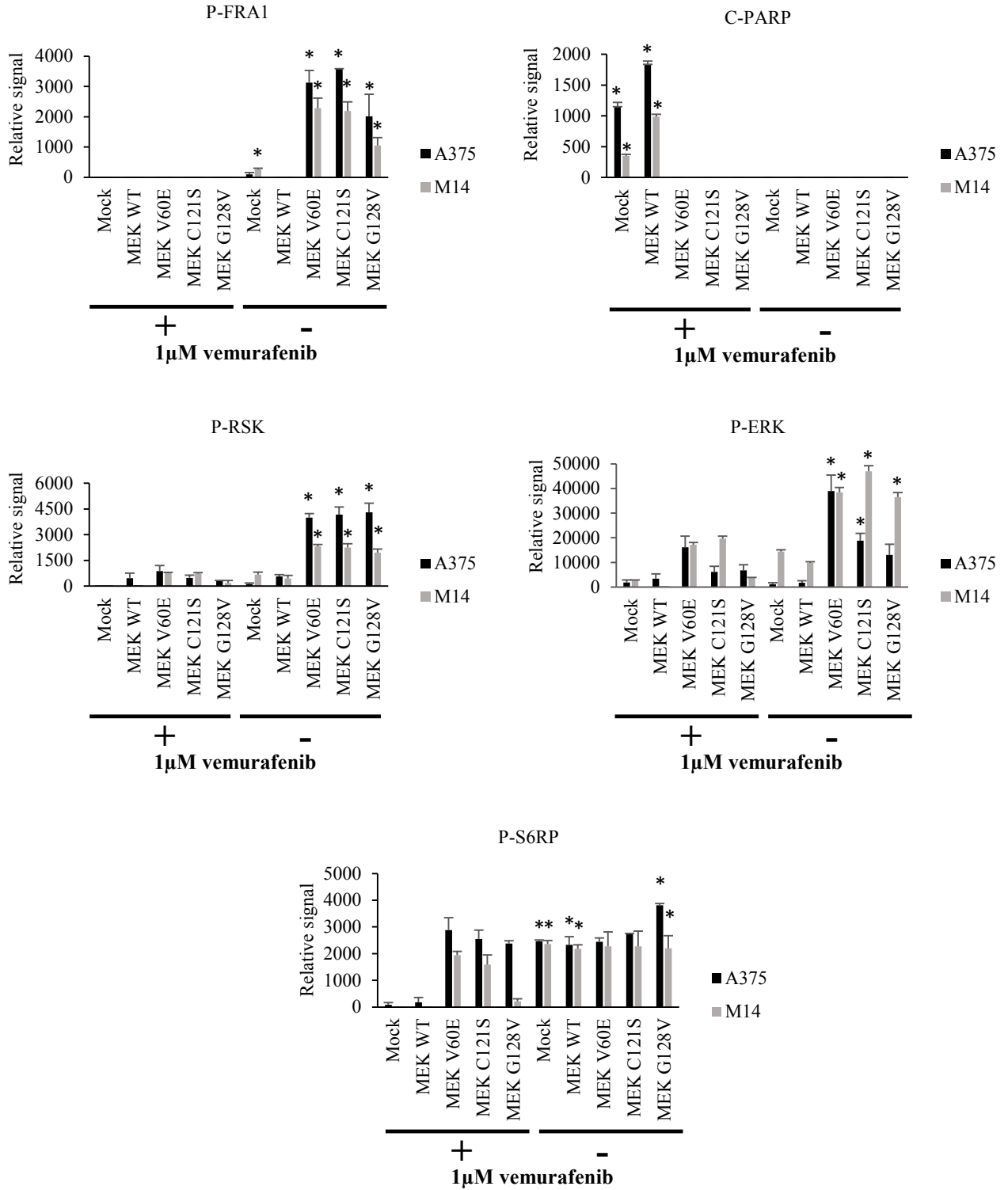

Supplement: Supplementary Figure 3 [file onc2016526x3.pdf]

# Supplementary Figure 4.

A

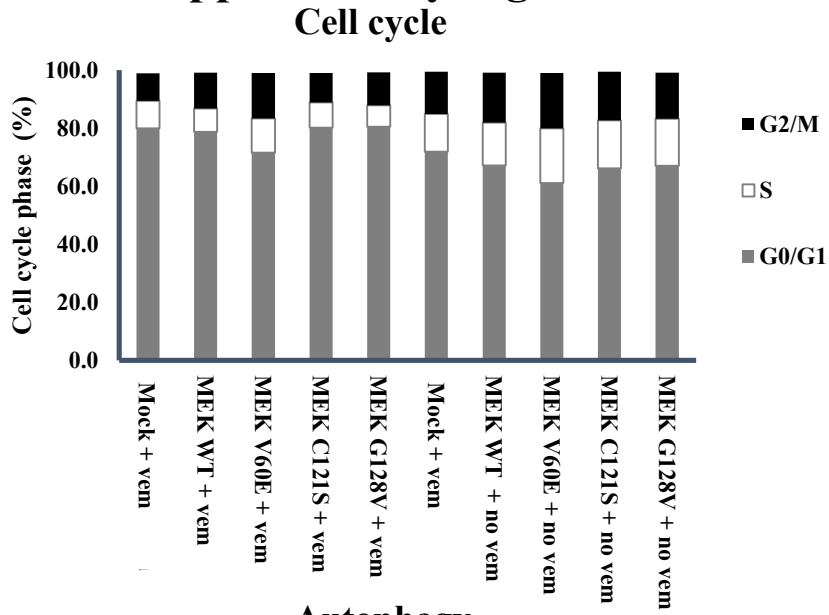

B

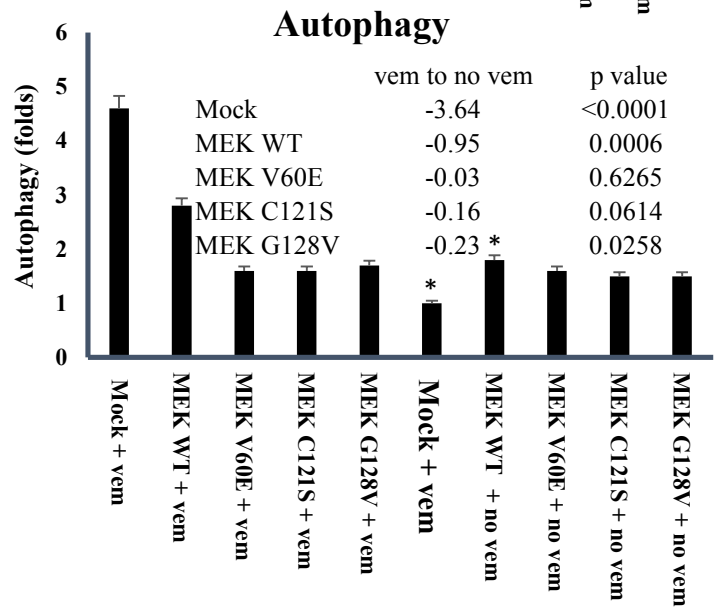

C

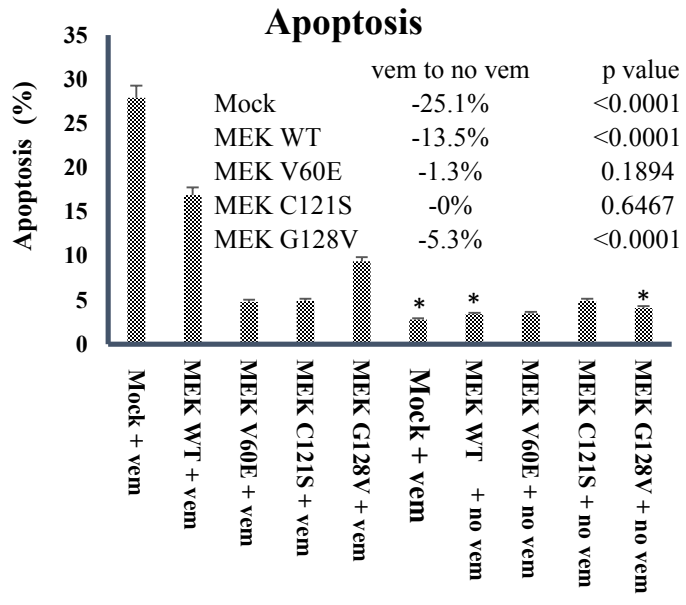

Supplement: Supplementary Figure 4 [file onc2016526x4.pdf]

# Supplementary Figure 5.

**A**

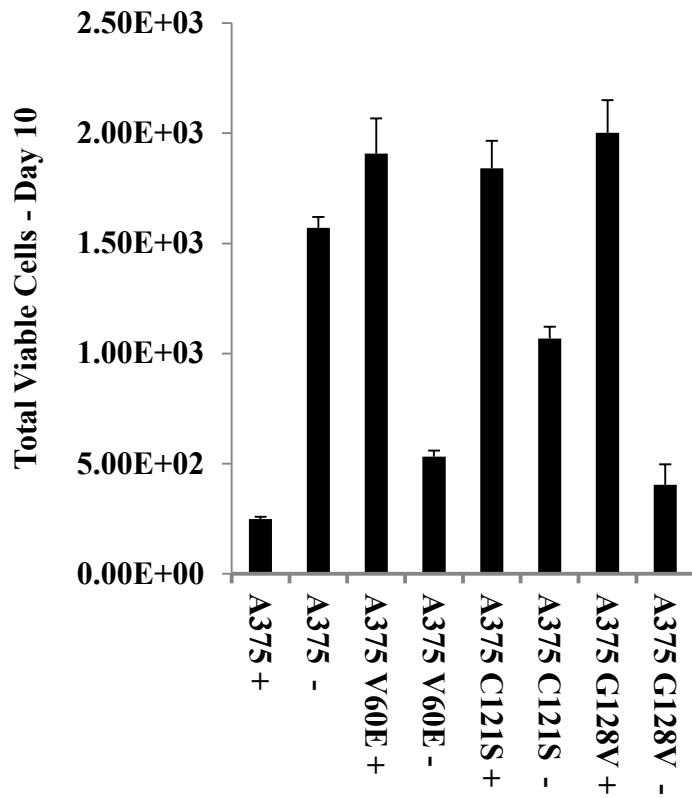

**C**

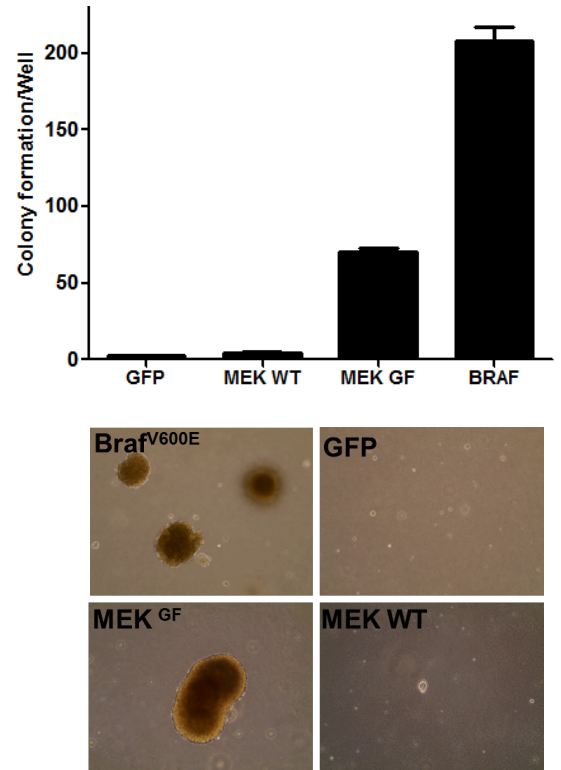

**B**

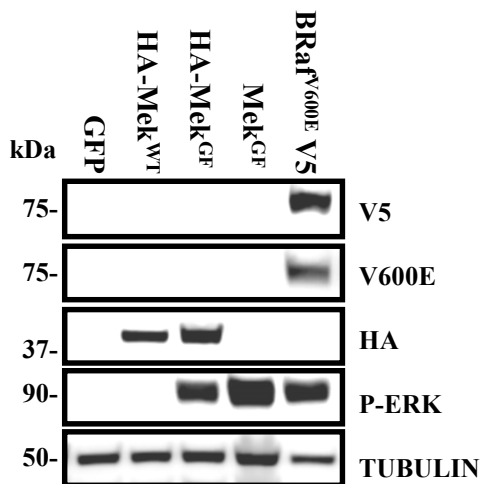

**D**

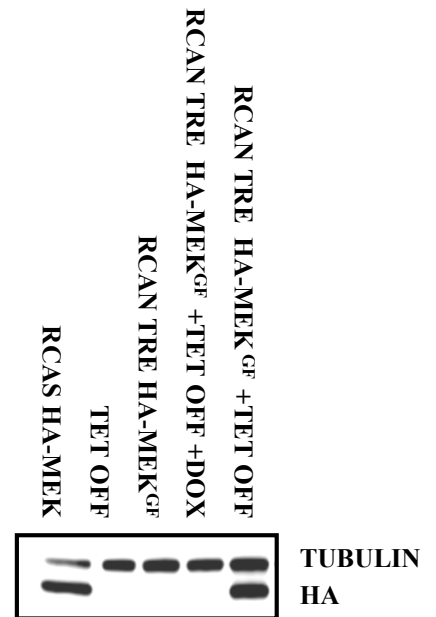

Supplement: Supplementary Figure 5 [file onc2016526x5.pdf]

# Supplementary Figure 6.

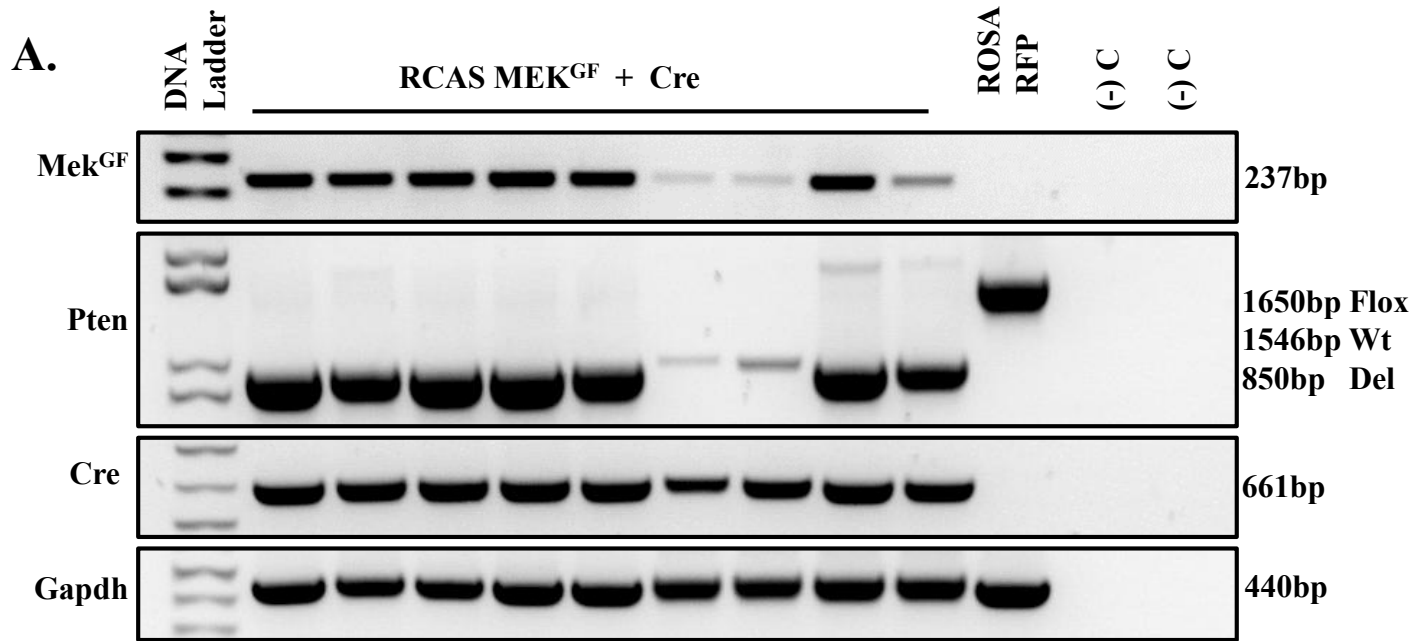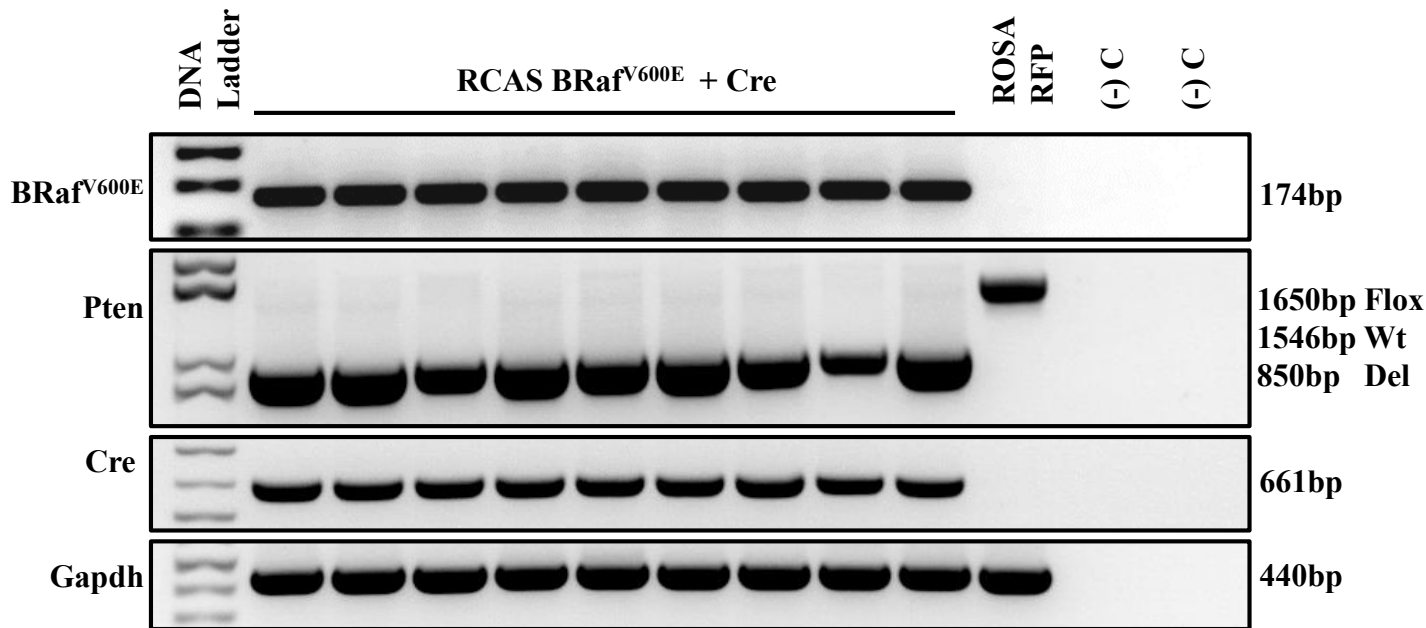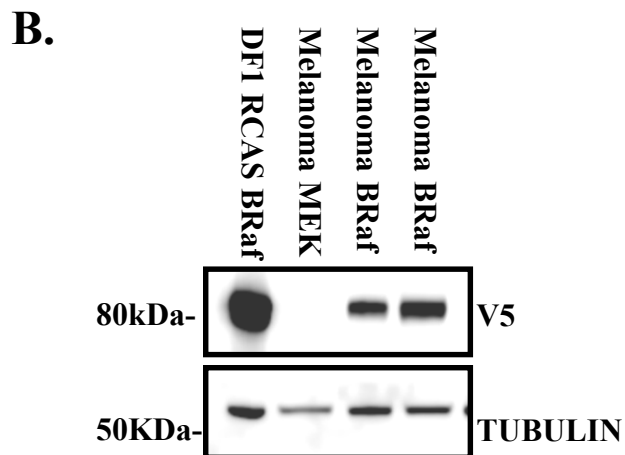

Supplement: Supplementary Figure 6 [file onc2016526x6.pdf]

Supplementary Figure 7.

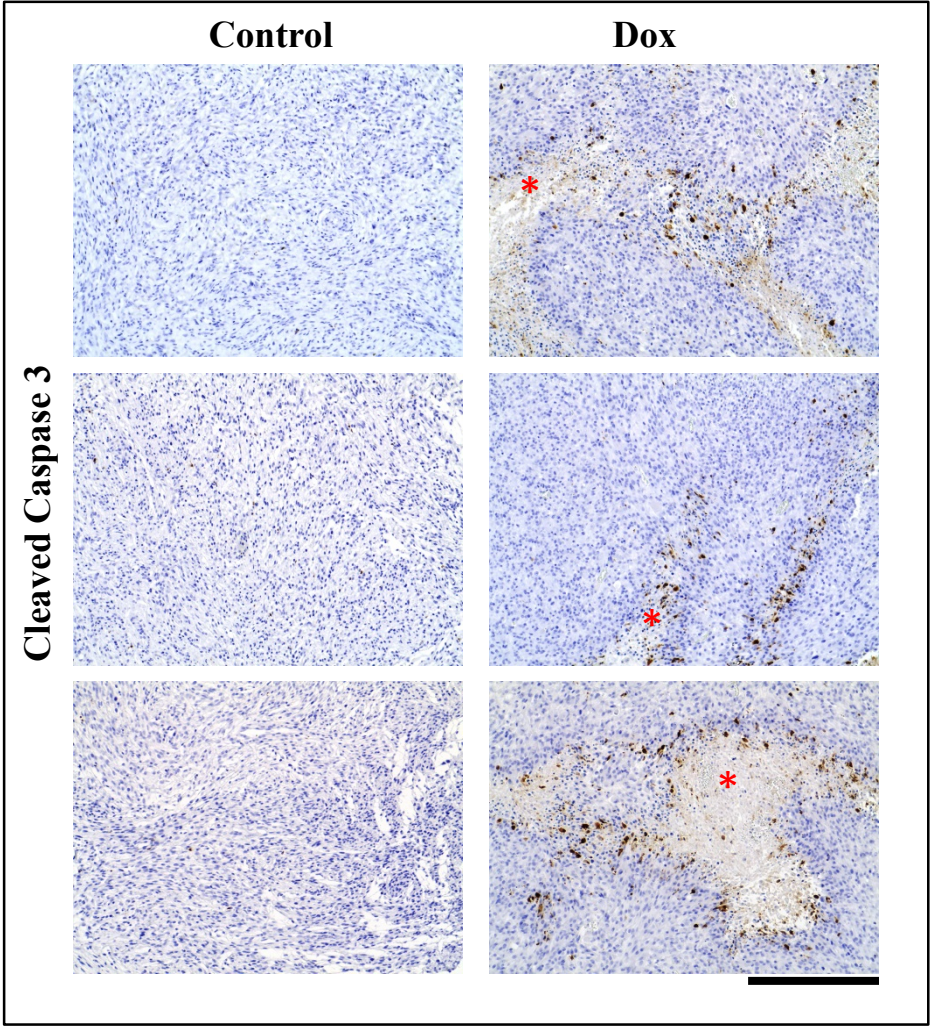

Supplement: Supplementary Figure 7 [file onc2016526x7.pdf]
